# Supplementary material for: In situ mechanical reinforcement of polymer hydrogels via metal-coordinated crosslink mineralization
Source: Nat Commun. 2021 Jan 28;12:667. doi: 10.1038/s41467-021-20953-7 (PMC7844223; doi:10.1038/s41467-021-20953-7)
Supplement: Supplementary file 1 — Supplementary Information [file 41467_2021_20953_MOESM1_ESM.pdf]

nature COMMUNICATIONS

Supplementary Information

In situ mechanical reinforcement of polymer hydrogels via metal-coordinated crosslink mineralization

By Sungjin Kim[1,†], Abigail U. Regitsky[1,†], Jake Song[1], Jan Ilavsky[2], Gareth H. McKinley[3] and Niels Holten-Andersen[1,]*

1. Department of Materials Science and Engineering
   Massachusetts Institute of Technology
   77 Massachusetts Avenue, Cambridge, MA 02139, United States of America
2. X-ray Science Division
   Advanced Photon Source
   Argonne National Laboratory, Lemont, IL 60439, United States of America
3. Department of Mechanical Engineering
   Massachusetts Institute of Technology
   77 Massachusetts Avenue, Cambridge, MA 02139, United States of America

† These authors contributed equally
* Corresponding Author: **holten@mit.edu**

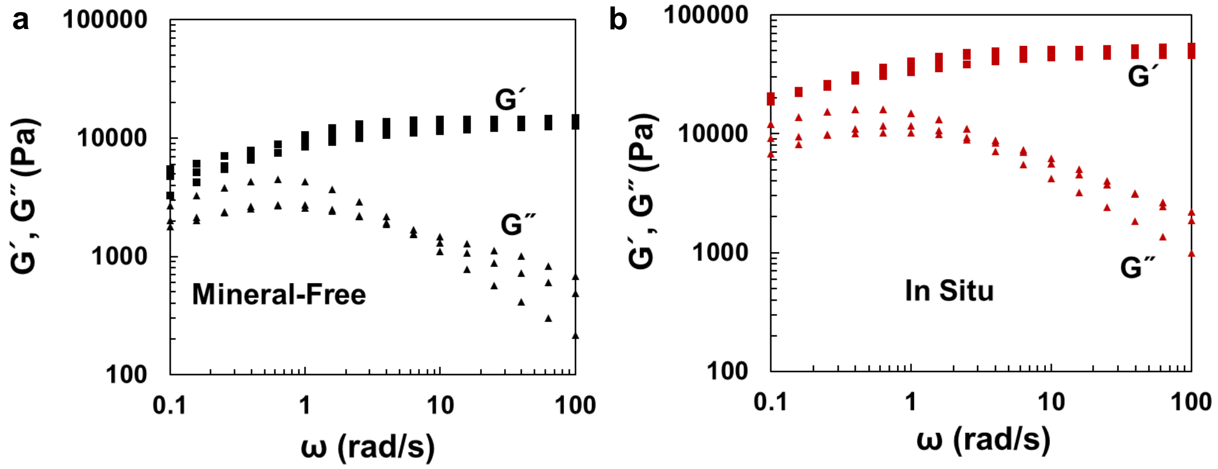

Supplementary Figure 1. Oscillatory frequency sweep tests of three separate specimen of (a) Mineral-Free (i.e., with no *in situ* mineralization) and (b) In Situ (i.e., after *in situ* mineralization) samples. The plateau modulus G_p increase more than three-fold upon *in situ* mineralization from G_p (Mineral-Free) = 14 ± 1 kPa to G_p (In Situ) = 49 ± 3 kPa. Storage modulus G' and loss modulus G'' are depicted with squares and triangles, respectively.

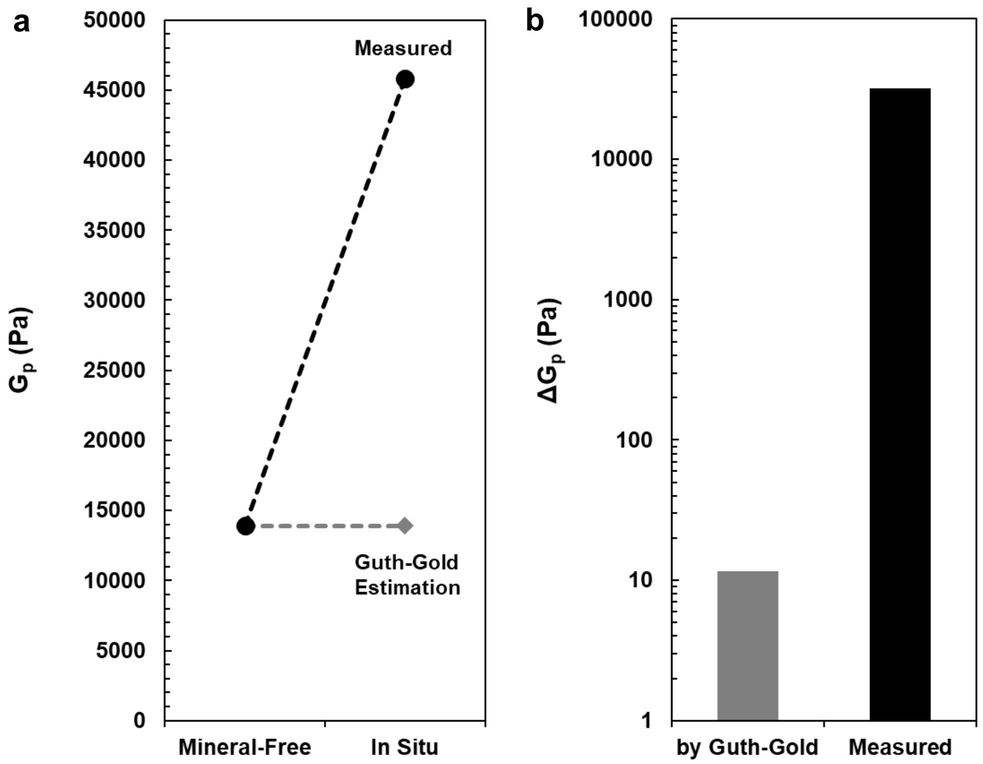

Supplementary Figure 2. (a) Comparison of measured G_p and estimated G_p of In Situ gel using Guth-Gold equation for non-interacting fillers in a rubber: $G_p = G_0(1 + 2.5\phi + 14.1\phi^2)$, where G_0 is the stiffness of the gel without fillers (i.e., G_p of the Mineral-Free gel) and ϕ is the estimated maximum volume fraction of filler in an In Situ gel. The volume fraction used in this estimate is an upper bound, assuming a 100% mineralization yield from the reactants put into the gel according to the reaction: $2Fe^{3+} + Fe^{2+} + 8OH^- \rightarrow Fe_3O_4 + 4\ H_2O$, resulting in 0.074 vol%. (b) The stiffness increase (ΔG_p) measured upon *in situ* mineralization is three orders of magnitude higher than that estimated by Guth-Gold theory for the incorporation of non-interacting particles in gels, which supports that the minerals formed in In Situ gels interact strongly with the matrix polymer network.

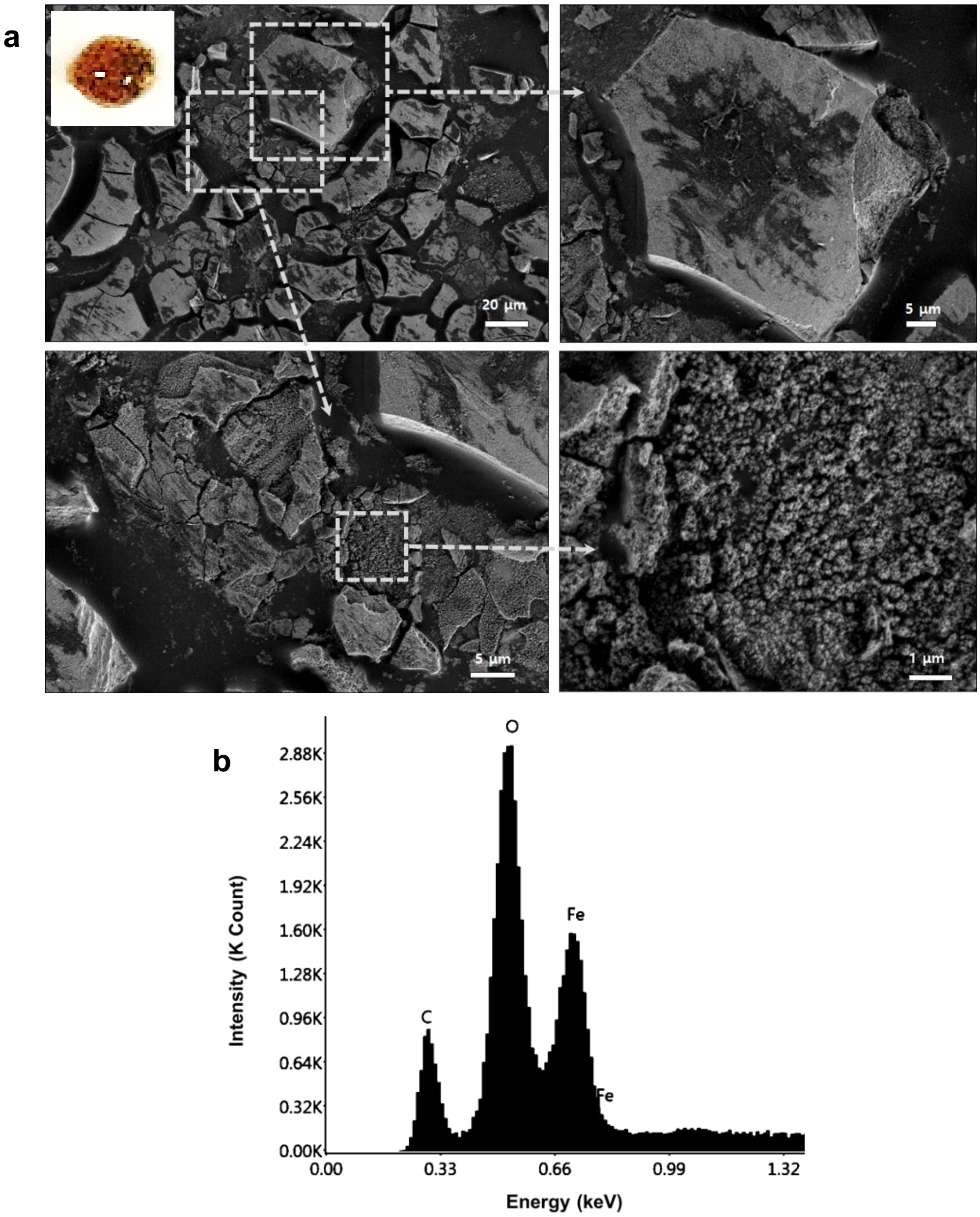

Supplementary Figure 3. (a) SEM images showing minerals formed without any polymer (from Mineral-Only samples, inset photo), washed with water and then dried in ambient conditions for 24 hours. In these polymer-free samples, iron oxide particles up to a size of ~ 50 μm can be observed (upper row). Nanoparticles, which typically agglomerate into larger particles[1], are also observed (lower row). (b) EDS shows the essential elements (i.e., Fe, O) of iron oxide minerals. The atomic percentage of Fe was 15%, and that of O was 64%. The 7% higher atomic percentage of O than that expected of Fe_3O_4 (i.e., 57%) is attributed to residual H_2O and/or the presence of other possible iron oxides of higher atomic fraction of O such as Fe_2O_3. We note that the Ex Situ gel samples are processed via first forming a dispersion of mineral precipitates (as shown in inset photo) by mixing all ingredients without 4cPEG, followed by later mixing with 4cPEG polymer solution.

TEM Image Analysis (Supplementary Figure 4-5)

We observed and classified small spherical iron oxide particles of a few nanometers (< 3.5 nm) in diameter in both In Situ ×1 and ×5 gels, while larger particles (> 3.5 nm) are observed only in In Situ ×5 gels. Thin, cylindrical particles are also observed in both samples. We consider the small and large spherical particles (labeled as "Small" and "Large", respectively) to be Fe_3O_4, whereas the rod-like, cylindrical particles (labeled as "Rods") are likely side products or intermediates, possibly $Fe(OH)_2$[2]. Quantitative image analysis of TEM micrographs revealed that the three types of particles display log-normal size distributions with calculated average radii of the Small and Large spherical particles of 1.4 (± 0.3) nm and 6.2 (± 2.5) nm, respectively, and an average length of 14.4 (± 5.3) nm and width of 3.3 (± 1.5) nm of the Rods. We note that extra-large species (labeled as "Aggregates", not included in the histograms), which appeared to be aggregates of the Large particles, were also observed in some TEM micrographs. Image analysis of these Aggregates revealed their average radius to be 23.4 (± 12) nm; a small sample size of aggregates led to a large standard deviation.

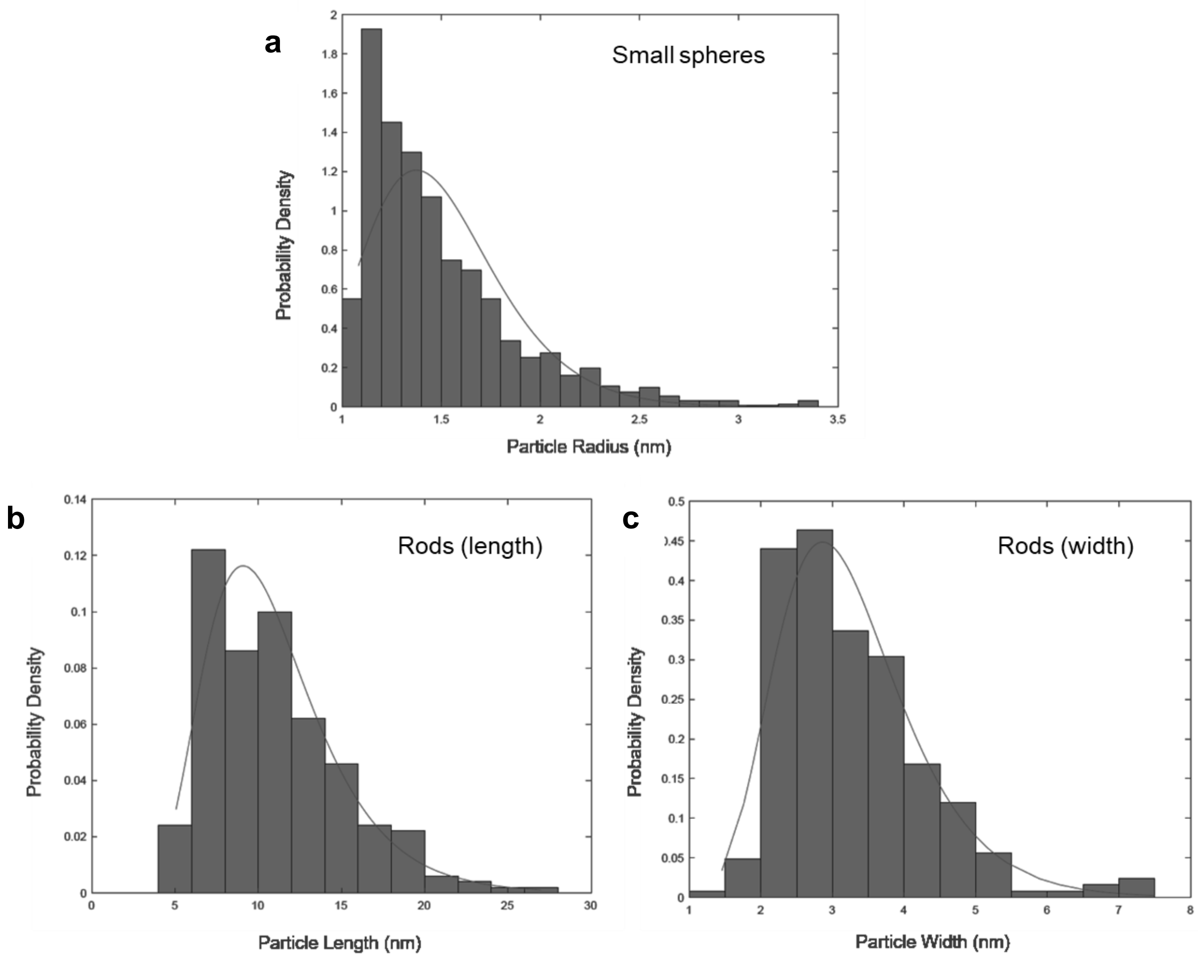

Supplementary Figure 4. Particle size histograms of In Situ × 1 gels of (a) spheres (Small), and (b) length and (c) width of cylinders (Rods), along with lognormal distribution fits (solid lines). In measuring the particles, we excluded instances below a certain pixel size to avoid counting noise, which caused the distributions to look cut off on the lower end.

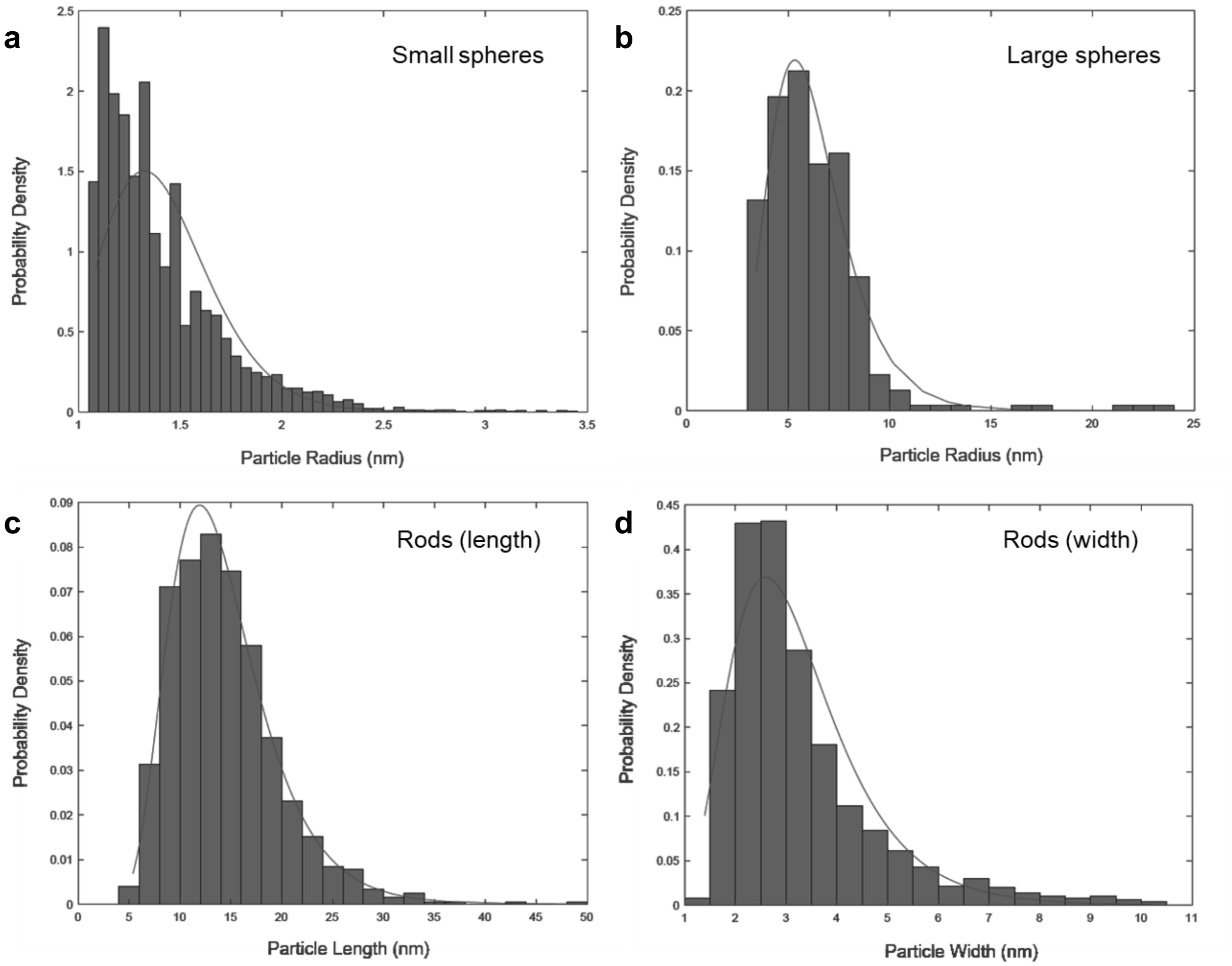

Supplementary Figure 5. Particle size histograms of In Situ × 5 gels of (a) Small spheres, (b) Large spheres, and (c) length and (d) width of Rods, along with lognormal distribution fits (solid lines). In measuring the particles, we excluded instances below a certain pixel size to avoid counting noise, which caused the distributions to look cut off on the lower end.

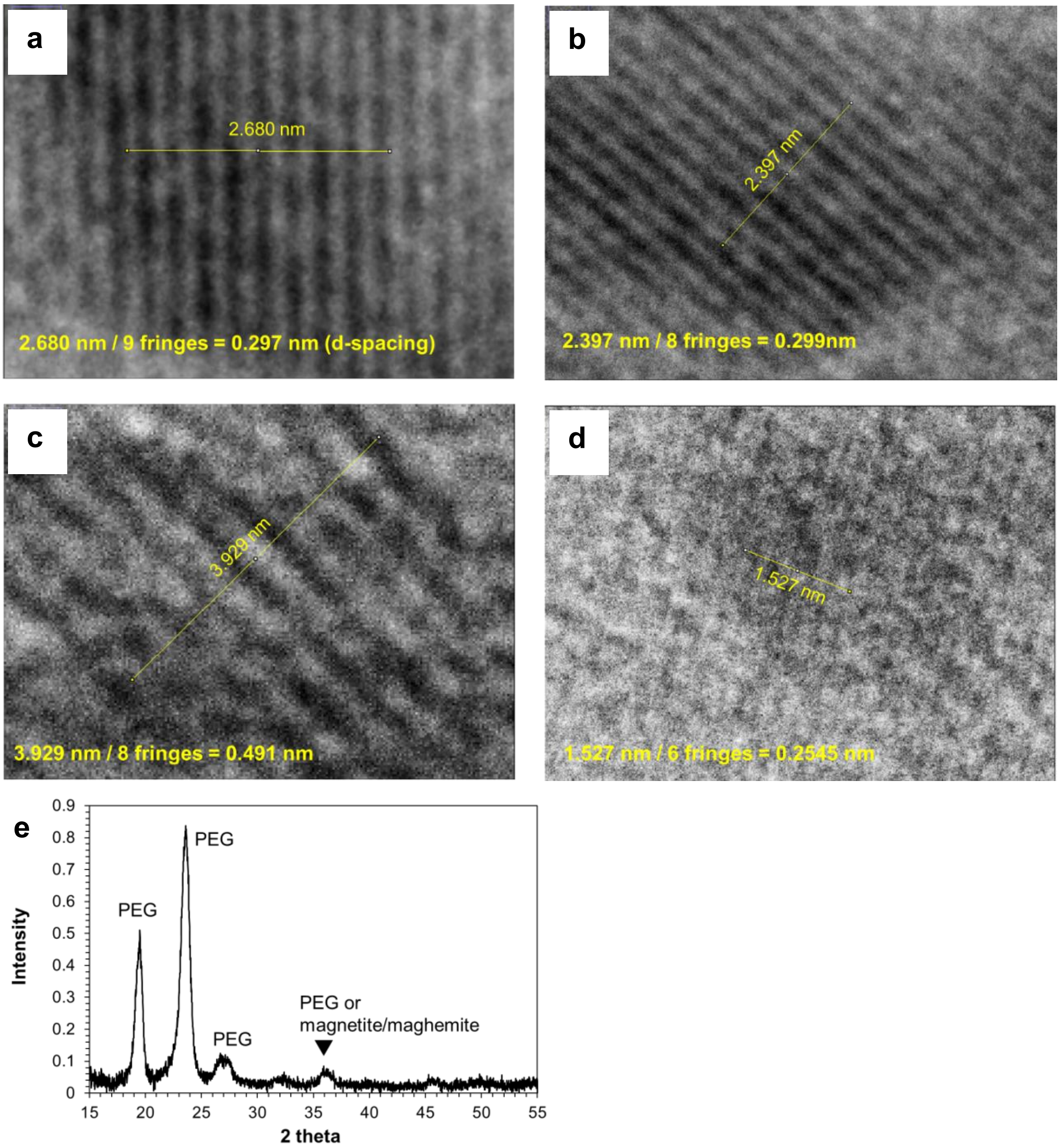

Supplementary Figure 6. Lattice spacing (D-spacings) of minerals (Aggregates) observed by HRTEM (a-d) and (e) XRD spectrum of an In Situ gel. The d-spacings observed are (a) 0.297 nm, (b) 0.299 nm, (c) 0.491 nm, and (d) 0.2545 nm. Note that the top two TEM images (a, b) are showing the particle in the inset of Figure 2c. These values match close to reported values: (a, b) 0.2967 nm, (c) 0.4852 nm and (d) 0.2532 nm for (220), (111), and (311) planes, respectively, of magnetites[3–6]. We note our observed d-spacing values are closer to those of magnetites than those of maghemites: 0.2953 nm, 0.2518 nm for (220) and (311) planes, respectively, while the d-spacing value for (111) plane of maghemite was unavailable in our references[3–7]. We, however, note that the maghemites could be also included as an impurity in our In Situ system seen from small trace characteristic Raman bands for maghemites (Figure 1g). (e) XRD pattern of In Situ gels. The most prominent peak for the (311) plane of magnetites or maghemites at 35.42° or 35.63°, respectively, coincides with a peak originating from the PEG polymer[8] and could therefore not be clearly distinguished given the small mineral particle fraction in our systems[8]. Hence, the iron oxide species were characterized by HRTEM and Raman spectroscopy.

Magnetic Analyses (Supplementary Figure 7-8)

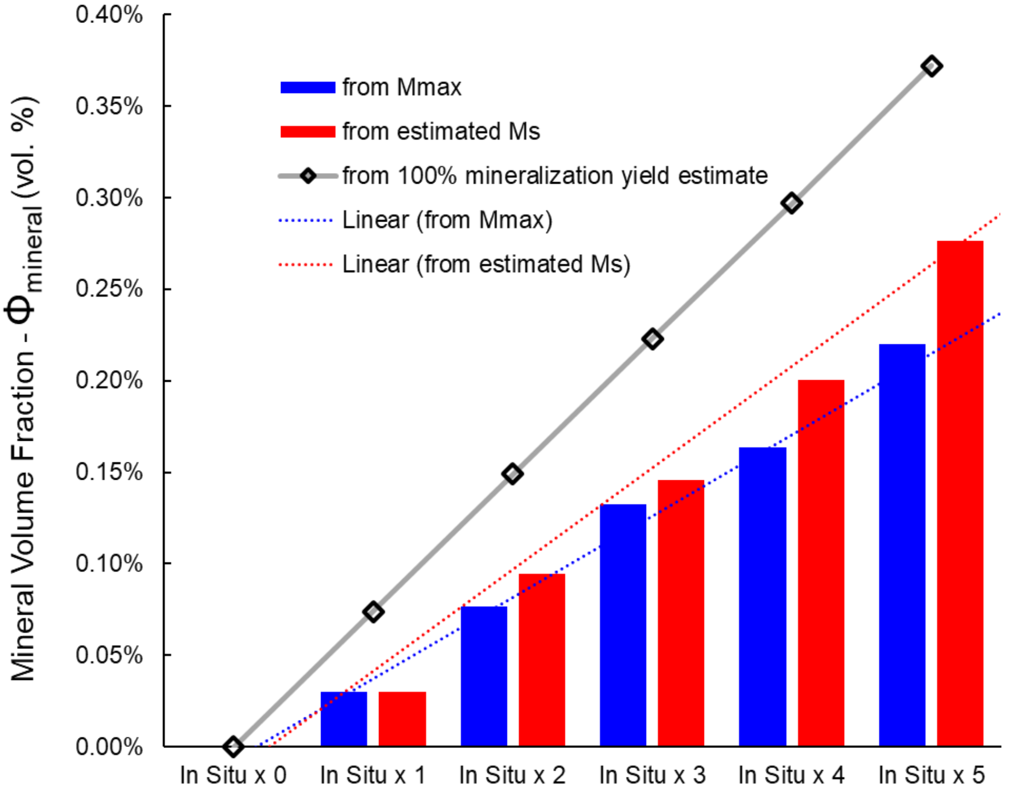

Supplementary Figure 7. The mineral volume fraction in the hydrogels (Φ_{magnetic}) calculated from the measured maximum magnetization (M_{max}, blue bars), estimated saturation magnetization (M_s, red bars), and the 100% mineralization yield estimate (black diamonds). Magnetization values were obtained from the VSM measurement on dry gels. The saturation magnetization (M_s) was approximated using the linear extrapolation of the 1/H vs M curves in the high magnetic field (H) region ($1/H < 2.05 \times 10^{-5}$ Oe^{-1}) for the In Situ × 2 – 5 that did not show a saturation under the measured H range (fitting data available in Supplementary Figure 8). The Φ_{magnetic} was calculated from the magnetization values as follows:

$$\Phi_{\text{magnetic}} = \frac{V_{mineral}}{V_{hydrogel}} = \frac{V_{mineral}}{V_{solvent} + V_{mineral}} = \frac{\frac{m_P}{\rho_P} \times \frac{M}{M_{s,pure}}}{V_{solvent} + \frac{m_P}{\rho_P} \times \frac{M}{M_{s,pure}}}$$

where $V_{mineral}$ is the volume of the mineral and $V_{hydrogel}$ is the volume of the whole hydrogel including the $V_{mineral}$. The m_P and ρ_P is the mass (2.5 mg) and the density of the polymer (1.0 - 1.2 g/mL), respectively. $V_{solvent}$ is the volume of solvent (i.e., water) of the hydrogel (20 μL). $M_{s,pure}$ is the saturation magnetization of pure magnetite (86 emu/g) and M is the measured magnetization of the dry gel samples. Note that the $V_{hydrogel} \approx V_{solvent}$ since $V_{solvent} \gg V_{mineral}$. The Φ_{magnetic} values derived from the magnetization data

were all consistently below those from the 100 % yield assumption, as expected, and showed a linear increase with repeating mineralization cycles (linear trends shown by dotted lines). In addition, the value of ~0.22 vol% and ~0.28 vol% obtained using the M_{max} and the estimated M_s, respectively, for the In Situ × 5 are reasonably close to the $\Phi_{microscopy}$ ~0.29 vol% calculated from the TEM image analysis. While there could be some product loss from the sample washing step, we note that the $\Phi_{magnetic}$ obtained from the magnetization analysis treats all minerals as magnetite, which thereby discounts any possible volume fraction of less magnetic side products or intermediates such as $Fe(OH)_2$ or maghemites in agreement with the slightly higher volume fraction obtained from TEM image analyses, which includes all minerals. We also note that the very small sized particles may have a much smaller number of domains and magneto-crystalline anisotropy, which can respond differently to the external field, causing the deviance in the magnetization. Furthermore, since we used dehydrated instead of hydrated gels to prevent sample volume change during the multi-hour measurement, it is likely that the dehydration shortened interparticle distances in the gel, which could possibly form extrinsic magnetic anisotropy thereby inducing spontaneous magnetism. However, based on the superparamagnetic behavior lacking a coercivity in our dehydrated gels, such events were negligible to affect the magnetic properties.

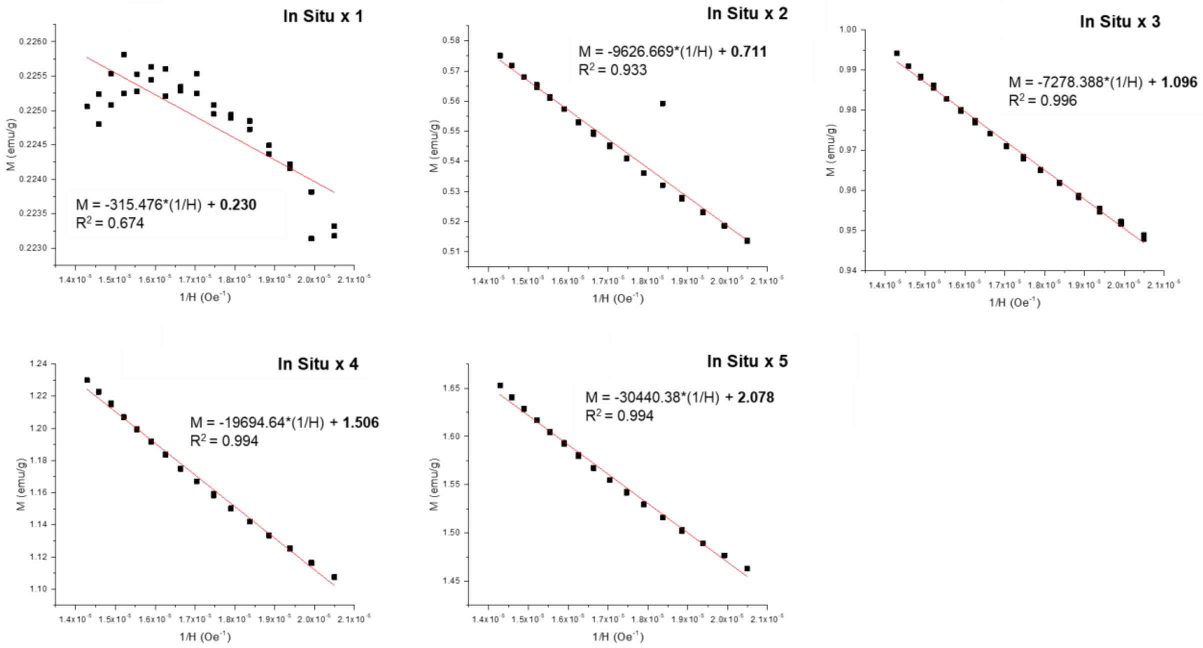

Supplementary Figure 8. The saturation magnetization (M_s) approximated by the linear extrapolation of the 1/H vs M curves in the high magnetic field (H) region ($1/H < 2.05 \times 10^{-5}$ Oe^{-1}) for all In Situ mineralized systems. The intercept of the fitted line can be estimated as the M_s where H goes to infinity. The data follows the linear fits in all the samples that did not display saturation under the measured H range (In Situ × 2 – 5). However, In Situ × 1 showed a visible saturation in the original H vs M curve.

SAXS Analyses and Modelling (Supplementary Figure 9-10)

A. Scattering Models

The combined model referenced in the main text combines scattering contributions from star polymers, small and large spheres, cylinders, and aggregates. All fitting procedures are performed on the Igor Pro software using the Irena package[9]. Intensity measurements can be generally modeled by the following relation:

$$I(q) = \varphi(\Delta\rho)^2 P(q) S(q) \tag{1}$$

Where *I(q)* is the overall scattering intensity, *P(q)* is the form factor, *S(q)* is the structure factor, $(\Delta\rho)^2$ is the X-ray scattering contrast and φ is the volume fraction. $(\Delta\rho)^2$ and φ are typically difficult to decouple from SAXS data without making explicit assumptions, and as such are treated here as a single scaling parameter, with the contrast held at $(\Delta\rho)^2 = 100 \times 10^{20} cm^{-4}$ across all form factors. Additionally, our data can be described explicitly through form factors alone, and thus *S(q)* is set to 1.

The scattering intensity for a sphere is thus described by:

$$I_{sphere}(q) = \frac{\varphi}{V}\left[3V\frac{\sin(qr) - qr\cos(qr)}{(qr)^3}\right]^2 \tag{2}$$

Where $V = \frac{4}{3}\pi r_{sphere}{}^3$ is the volume of the scattering sphere and r_{sphere} is the radius of the sphere[10]. Both the small and large sphere models adopt this equation.

The scattering intensity for a cylinder (rod) is described by:

$$I_{cylinder}(q) = \frac{\varphi}{V}\int_0^{\pi/2} F^2 \sin\alpha \, d\alpha$$

$$F = 2V\frac{\sin\left(\frac{q}{2}L_{cylinder}\cos\alpha\right)}{\frac{q}{2}L_{cylinder}\cos\alpha}\frac{J_1(qR\sin\alpha)}{qR\sin\alpha} \tag{3}$$

Where $V = L_{cylinder}\pi r_{cylinder}{}^2$ is the volume of the cylinder, $L_{cylinder}$ and $r_{cylinder}$ are the length and radius of the cylinders respectively, α is the angle between cylinder axes, and J_1 is the first-order Bessel function.

Lastly, the scattering intensity for aggregates is captured by the Beaucage model[11]:

$$I_{aggregates}(q) = G_i e^{-q^2 R_g{}^2/3} + e^{-q^2 R_{gcutoff}{}^2/3} B_i\left[\frac{\left(\mathrm{erf}(qR_g/\sqrt{6})\right)^3}{q}\right]^{P_i} \tag{4}$$

Where G_i captures the scaling of the Guinier region described by R_g, the radius of gyration of the correlation element – made of aggregations of primary populations – and B_i captures

the scaling of the power-law region scaled by a power of P_i. A Beaucage model is used here as it is routinely used for modeling of scattering from fractal aggregates (see Fig. 2c). We note that no volume fraction parameters are explicitly stated here as the volume is accounted for in the Guinier parameter G_i.

The combined model is a summation of the three scattering models: $I_{total}(q) = (I_{small\ spheres}(q) + I_{large\ spheres}(q)) + I_{cylinder}(q) + I_{aggregates}(q)$.

B. Model Parameters and Fit Results Discussion

The size parameters for the small and large sphere models, cylinder model, and the aggregate model in the combined fit are obtained *a priori* directly from TEM image analyses (see Supplementary Figure 4, 5). The only fitting parameter in the combined fit is φ (which scales the combined contribution of volume fraction and the contrast, $\varphi(\Delta\rho)^2$ in Supplementary Equation 1, as $\Delta\rho$ is set to a fixed value discussed above). We note that the estimated volume fraction of the minerals after fitting are in the same order of magnitude as the TEM predictions, despite the convolution in intensities due to the polymer scaffold and gel thickness effects.

We utilize two populations for the spheres, corresponding to the two populations identified in the TEM images. Single populations are adopted for the rods (cylinders) and aggregates. Mean values for $r_{small-sphere} \sim 1.4$ nm, $r_{large-sphere} \sim 6.3$ nm, $r_{cylinder} \sim 3.3$ mm and $L_{cylinder} \sim 14.3$ nm are used to construct the fits. Moreover, in accordance with the TEM statistics, we apply polydispersity to our size parameters (such that $r_{sphere} \to \langle r_{sphere} \rangle$) and follow the log-normal distributions identified in the TEM images for the spheres and the rods. The standard deviation σ is obtained from TEM statistics for the sphere and cylinder models. For the aggregates, we set $R_{g_{cutoff}} = 6.3$ nm as the aggregates consist of clusters of the large-spheres (Fig. 2C), and obtain G_i, R_g and P_i through fitting. B_i is calculated from these values using assumptions of the Guinier-Porod model. This process yields $R_g = 17.89$ nm for the aggregate radius which is in good agreement with TEM (Fig. 2C).

All fitting parameters are listed in Supplementary Table 1, and a compilation of the individual fits as well as the combined fit is shown in Supplementary Figure 1_scattering.

Supplementary Table 1. Fitting parameters used to generate the combined model.

Small-sphere	Large-sphere	Rods (cylinders)	Aggregates
Vol. Fraction = 0.014<br>$r_{sphere} = 1.4$ nm<br>$\sigma = 0.20$	Vol. Fraction = 0.001<br>$r_{sphere} = 6.3$ nm<br>$\sigma = 0.34$	Vol. Fraction = 0.001<br>$r_{cylinder} = 3.3$ nm<br>$L_{cylinder} = 14.3$ nm<br>$\sigma = 0.34$	$G_i = 20.4$<br>$R_g = 17.89$ nm<br>$B_i = 3.4 \times 10^{-4}$<br>$Pi = 1.844$<br>$R_{g_{cutoff}} = 6.3$ nm

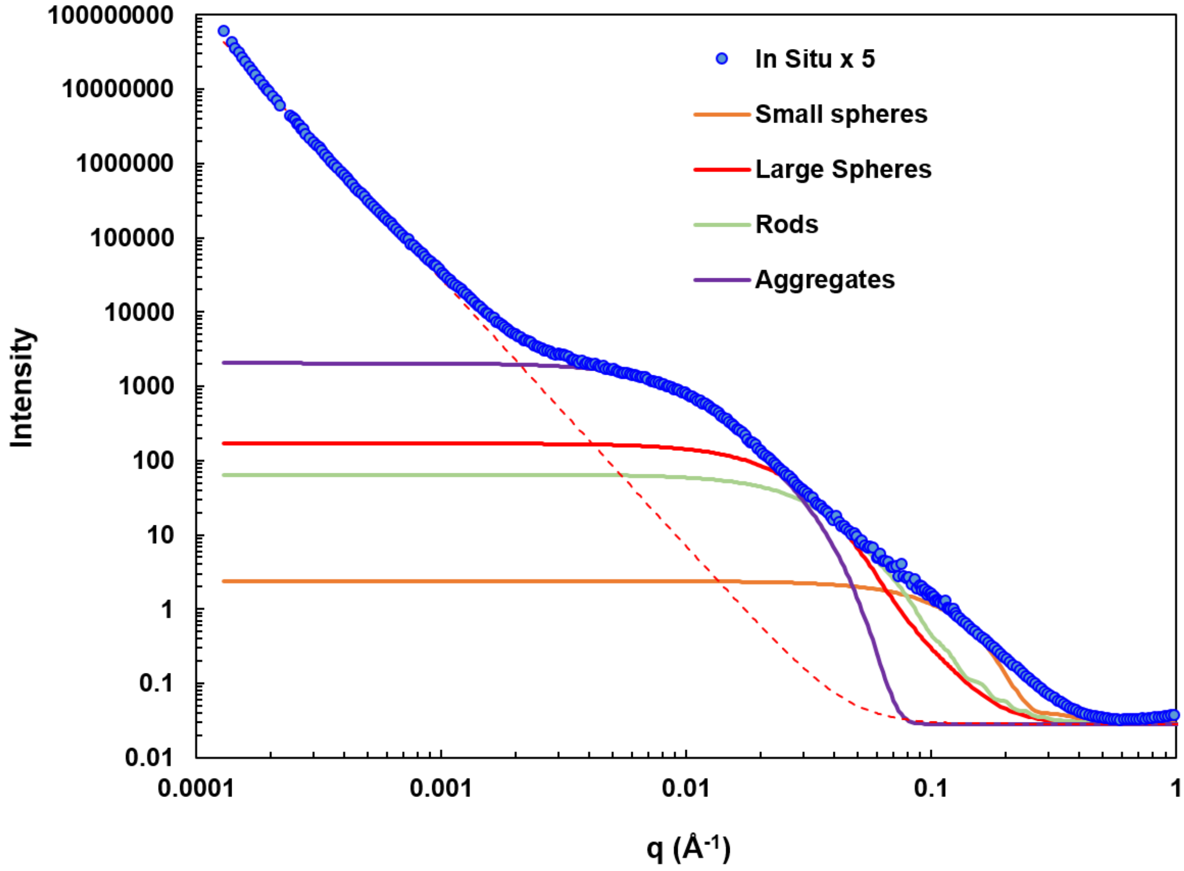

Supplementary Figure 9. Deconstructed view of the combined model, illustrating contributions of the two sphere (small, large) models, cylinder (rod) model, and aggregate (Beaucage) model. Scale parameters such as volume fractions are adjusted to vertically translate and thus overlay the individual models to the *in situ* scattering data. The dashed line indicates the power-law scaling from the macrostructure scattering at low q.

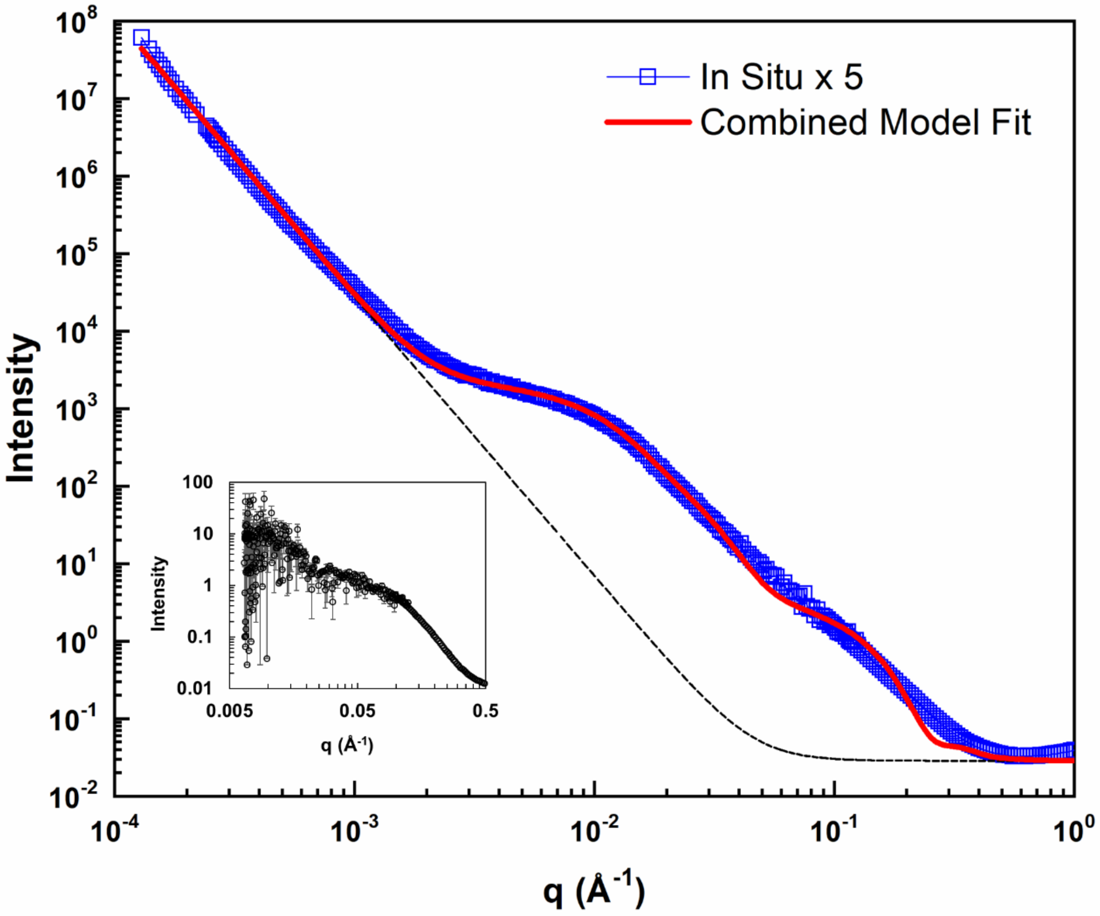

Supplementary Figure 10. SAXS scattering result for the Mineral-Free gel (black, inset) compared with In Situ × 5 gel. Mineral-Free sample (black inset) scattering is much weaker and the dominating feature is Guinier knee around $q \sim 0.05$ Å^{-1} with plateau intensity at lower q values. In situ × 5 sample has additional, significantly stronger scattering extending into much lower q-values.

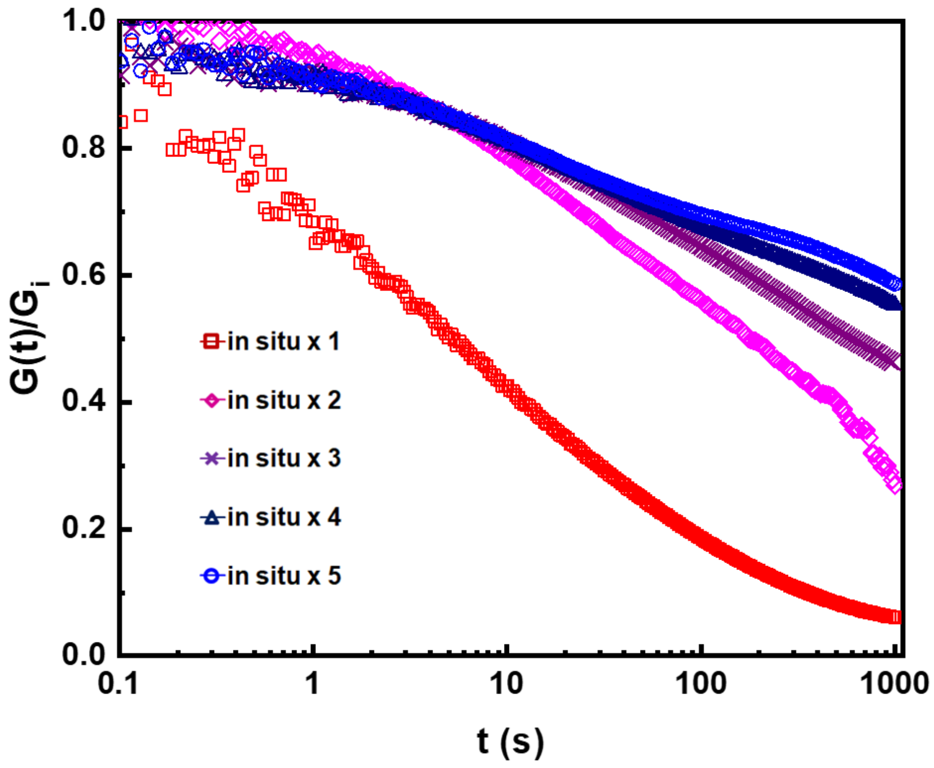

Supplementary Figure 11. Step-strain (10 % strain) curves of In Situ mineralized samples (i.e., In Situ × 1 - 5). As the hydrogel goes through more mineralization cycles, it relaxes slower.

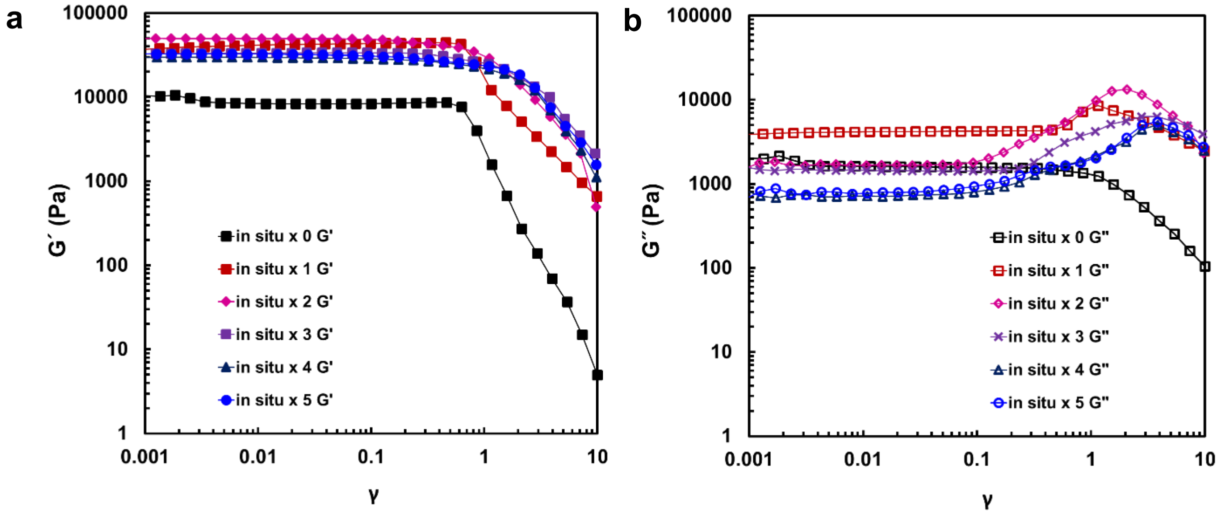

Supplementary Figure 12. Strain sweep (at constant ω of 10 rad/s) showing (a) G′ and (b) G″ of Mineral-Free (i.e., In Situ ×0) and In Situ mineralized samples (i.e., In Situ ×1 - 5) upon strain from 0.001 to 10. The mineralized samples show the Payne effect as in filled rubbers[12,13] – the G′ starts to decrease above 0.1 strain amplitude and the G″ shows a maximum peak likely due to energy dissipation by deformation-induced breakage of the mineral-catechol interfacial bonds in the network upon failure. Note that the energy dissipation peak is absent in the Mineral-Free gel. In addition, the G″ peaks shift right with increasing mineralization, indicating the more mineralized networks are dissipating energy at higher strain.

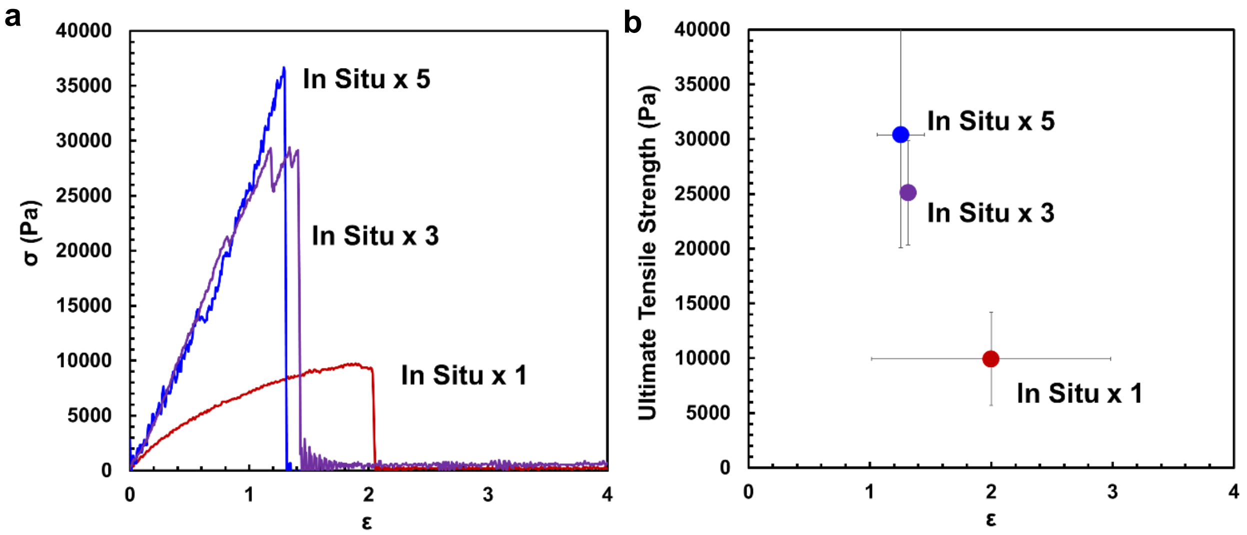

Supplementary Figure 13. Additional mineralization causes an increase in tensile strength. (a) Representative stress(σ)-strain(ε) curves for In Situ ×1 (red), ×3 (purple) and ×5 (blue). (b) Average ultimate tensile strength (UTS, dots) from each curve. The three individual specimens for each condition (error bars indicate standard deviations) were prepared as dogbone-shapes with 0.5× overall concentration of the samples used for other characterizations to ensure reliably controlled sample preparations in the dogbone-shaped mold, since gelation was too quick to controllably form uniform specimens if we used 1× concentration. As the mineralization is increased, higher UTS is clearly observed. The In Situ × 1 displays more ductile and less linear behavior compared with further mineralized gels. The trends in the strength correspond well with the LAOS observation (Figure 2h). Note that the Mineral-Free hydrogel was too soft to characterize reliably through tensile tests.

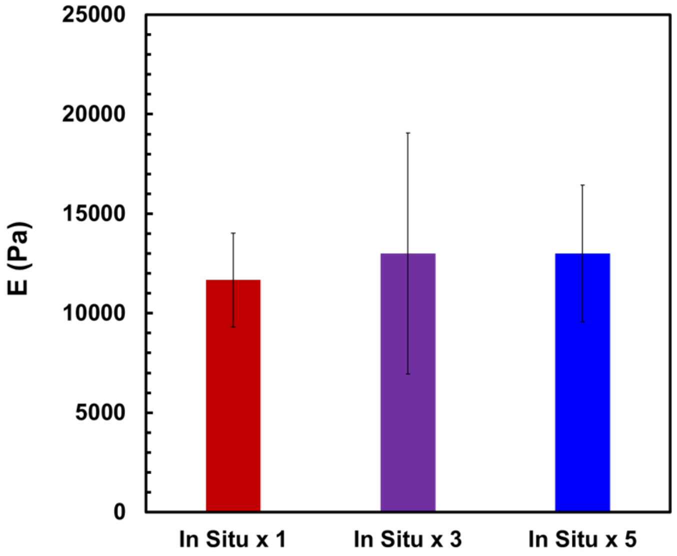

Supplementary Figure 14. Young's moduli (E) obtained from the tensile tests on three independent samples for each condition (error bars indicate standard deviations). Moduli were calculated at 0.1 strain in the linear region. As we have observed from G_p in rheological tests, the moduli after the first mineralization cycle do not differ significantly.

**Specimens**	**G_p (Pa)**	**ΔG_p (Pa) by mineral-incorporation**
Fe^{3+}-catechol gels[14]	15000	NA
Conventional NP gels[14]	10000	- 5000
In Situ × 0 (Mineral-Free)	13746 (± 939)	NA
In Situ × 1	48753 (± 3634)	+ 35007 (± 4136)
In Situ × 2	48923 (± 2993)	+ 35177 (± 3289)
In Situ × 3	50214 (± 1232)	+ 36467 (± 1779)
In Situ × 4	49899 (± 1071)	+ 36153 (± 1862)
In Situ × 5	48813 (± 429)	+ 35066 (± 1291)

Supplementary Table 2. The values of G_p and change in G_p by mineral-incorporation where $\Delta G_p = G_p$(with minerals) - G_p(without minerals). In the previous report by Li et al., nanoparticle gels (Conventional NP gels) are composed of the same polymer assembled with pre-synthesized nanoparticles of similar size (5-15 nm) as the *in situ* grown particles in this study and these gels were compared with their corresponding mineral-free counterpart (Fe^{3+}-catechol gels) reported in that same study[14]. Note that the 1.25× adjustment was processed to the approximate G_p values of the samples (100 mg/mL polymer concentration) in this reference[14] to match the polymer concentration (125 mg/mL) used in our work.

Reinforcement Mechanism and Rubber Elasticity Calculation (Supplementary Figure 15-16)

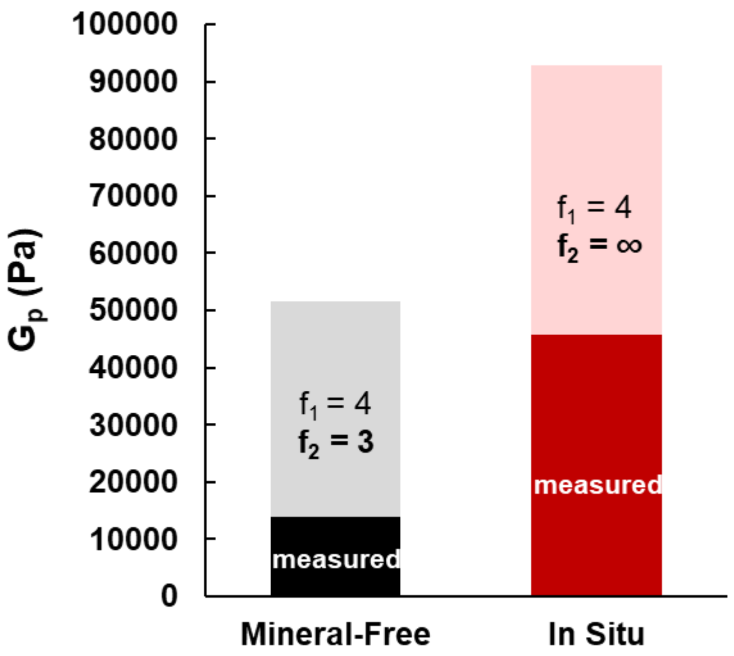

Supplementary Figure 15. G_p measured (dark-colored) and calculated by the rubber elasticity theory (dark-colored + light-colored) corresponding to the Mineral-Free system and In Situ system.

For calculating affine rubber elasticity of gels, we used the following equation:

$$G^{'} \approx G = \frac{\rho RT}{M_x}$$

where $\rho =$ 0.125 gcm^{-3} (the weight concentration of our gel), $R = 8.3145 \times 10^6$ cm^3 Pa K^{-1} mol^{-1} (ideal gas constant), $T = 298$ K (ambient temperature) and $M_x \approx 2500$ gmol^{-1} (molecular weight between crosslink junctions, i.e., average 1 arm length of our 10kDa 4-arm-PEG backbone polymer). Plugging in these values for 4-PEG gives G = 123886 Pa.

To account for the existence of crosslinks with different functionalities (f) in gels, we used phantom network theory as follows:

$$G^{'} \approx G = \frac{\rho RT}{M_x}\frac{(f-2)}{f} \Rightarrow \frac{\rho RT}{M_x}\left(1 - \frac{1}{f_1} - \frac{1}{f_2}\right)$$

where the modification of $\frac{(f-2)}{f} \rightarrow \left(1 - \frac{1}{f_1} - \frac{1}{f_2}\right)$ accounts for the different branch functionalities[15,16]. Hence, the phantom network of 4-arm-PEG ($f_1 = 4$) crosslinked by *tris*-coordination ($f_2 = 3$) gives 51619 Pa. Thus, our Mineral-Free gels with measured G_p ~ 14000 Pa is ~27 % of the stiffness expected for a perfect *tris*-coordinated 4-arm network, which is attributed to the existence of di-catechol covalent crosslinks ($f = 2$) in our case and elastically inactive defects as commonly observed in other studies of catecholic

networks[14,17,18]. Here, we consider that the metal-coordinate crosslinking efficiency itself is not 100 % regardless of the covalent crosslinks, leaving many ligands elastically inactive, since the G_p of mineral-free Fe:catechol=1:3 gels with limited covalent crosslinking (Supplementary Figure 17) or mineral-free Ni/Cu:histidine=1:2 gels without any covalent crosslinks (Figure 4) are all below the 51619 or 30912 Pa, respectively estimated for a perfect *tris*- or *bis*-coordinated 4-arm network.

If we consider a 4-arm network ($f_1 = 4$) crosslinked by infinite functionality ($f_2 \rightarrow \infty$), we get 92915 Pa from the calculation, which is theoretically the maximum modulus expected for a 4-arm phantom network crosslinked with heterogeneous functionalities. Thus, the G_p ~ 45000 Pa of In Situ gel is ~50% of the theoretical maximum modulus. It suggests that the fraction of the elastically active chains is increased in In Situ gels compared with Mineral-Free gels considering that the actual functionality cannot be infinite.

We also note another possible interpretation could be that the increase in the functionality solely resulted in the increase in G_p as the contribution of defects impairing the elasticity becomes less important in high functionality gels[15]. In this case, assuming the ~27% crosslinking efficiency of Mineral-Free gels stays the same in In Situ gels, the modulus expected for In Situ phantom network is ~25000 Pa, i.e., ~27% of 92915 Pa. Hence, this interpretation suggests that the measured G_p ~ 45000 Pa of In Situ gels are in the regime of affine network prediction.

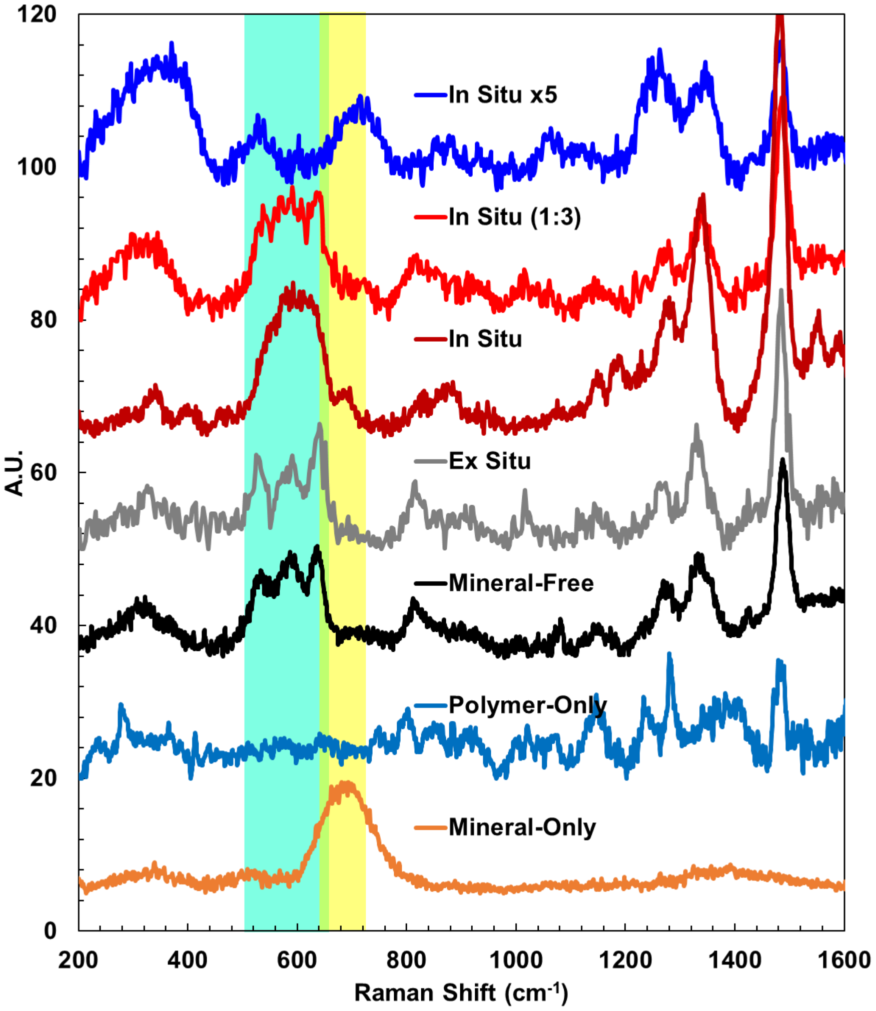

Supplementary Figure 16. Raman Spectra of all Fe and 4cPEG samples. The signal around ~680 cm^{-1} attributed to the iron oxides[19,20] (highlighted in yellow) is observed in Mineral-Only (orange), Ex Situ (gray), In Situ (dark red), In Situ (1:3) (red), and In Situ × 5 (blue) sample. The triplet peaks around ~530 to ~650 cm^{-1} attributed to O-Fe^{3+} coordination between the catechol and Fe^{3+} ions (highlighted in green) in high pH (Mineral-Free)[17] broadens in In Situ (i.e., In Situ × 1) and broadens further to nearly flatten out in In Situ × 5. This phenomenon of broadening is in correspondence with the transition of Fe^{3+}-catechol coordinate gels to Fe_3O_4 nanoparticle-catechol gels reported in the previous work[14]. Note that the In Situ (1:3) shows a lower degree of the broadening compared with the In Situ, indicating lesser mineral formation at the crosslink sites (Please see Supplementary Figure 17 for the details on this sample). This is in accordance with relatively lower increase in G_p in the In Situ (1:3) compared to the regular In Situ (Supplementary Figure 17).

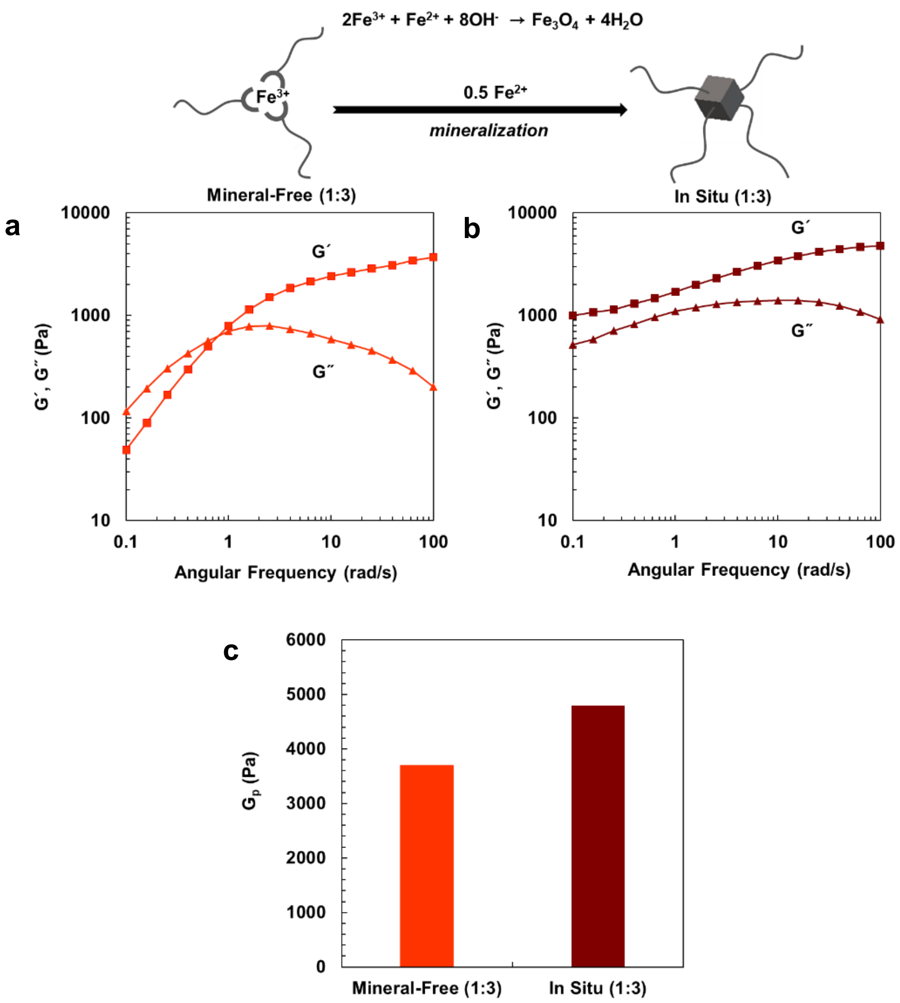

Supplementary Figure 17. Rheological frequency sweep profiles of (a) Fe^{3+}:catechol=1:3 gel (Mineral-Free (1:3)) and (b) with 0.5 molar equivalent of Fe^{2+} to Fe^{3+} added to induce mineralization (In Situ (1:3)). (c) G_p of Mineral-Free (1:3) and In Situ (1:3). The increase in G_p from Mineral-Free (1:3) to In Situ (1:3) is over 350 times higher than that estimated by the Guth-Gold equation (Supplementary Figure 2) for non-interacting particles assuming complete mineralization (i.e., 0.037 vol% of minerals). This increase in G_p by mineralization is relatively low compared to the Fe^{3+}:catechol=3:3 case (In Situ, Figure 1, Supplementary Figure 2), but the In Situ (1:3) gel still shows solid-like behavior compared to Mineral-Free (1:3), evidenced by $G' > G''$ at all measured frequencies. The low concentration of Fe^{3+} induce an insignificant level of catechol-oxidation and thereby a low level of catecholic covalent crosslinks in the network. These observations thus support that the *in situ* mineralization stiffen the network, regardless of the fraction of catecholic covalent crosslinks in the system (e.g., ~33 wt % for Mineral-Free gels based on estimates of the gel mass fraction after dissolving out the transient metal-coordinate crosslinked fraction of the gel network as reported by S. Kim et al.[21])."

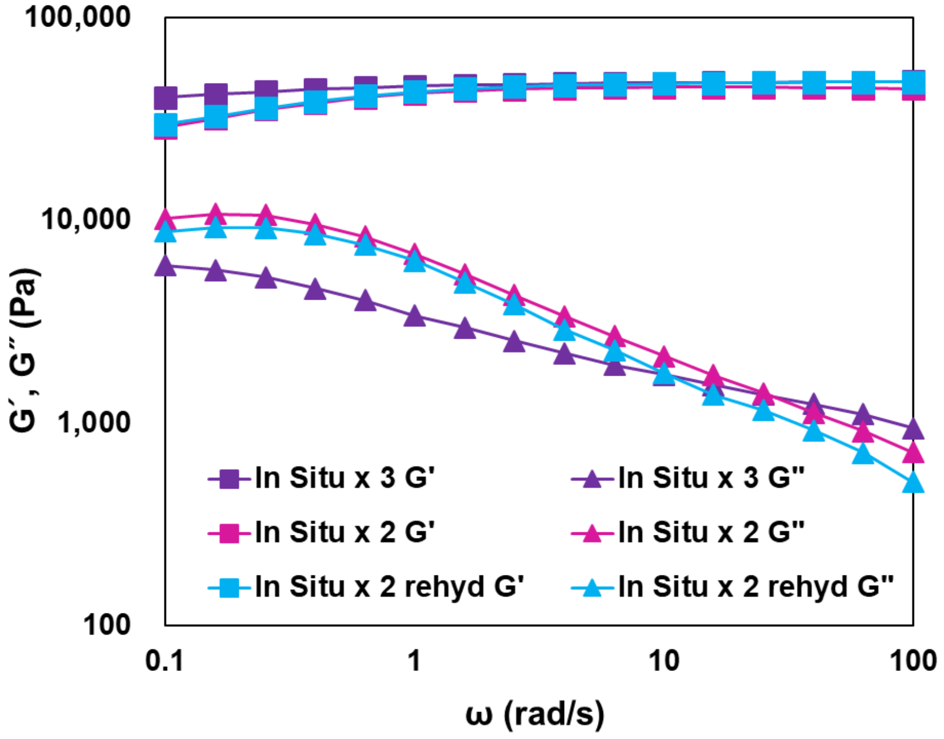

Supplementary Figure 18. Comparison of viscoelastic properties measured by rheological frequency sweep of In Situ × 2 (magenta), In Situ × 3 (purple) and In Situ × 2 rehyd (skyblue) that went through free dehydration over 24 hours in air then rehydrated with the equivalent original volume. The difference caused by dehydration is negligible (i.e., In Situ × 2 vs In Situ × 2 rehyd) compared to that caused by an additional mineralization cycle (i.e., In Situ × 2 vs In Situ × 3), suggesting that the dehydration is not the major cause of the mineralization to induce the solidification of the material.

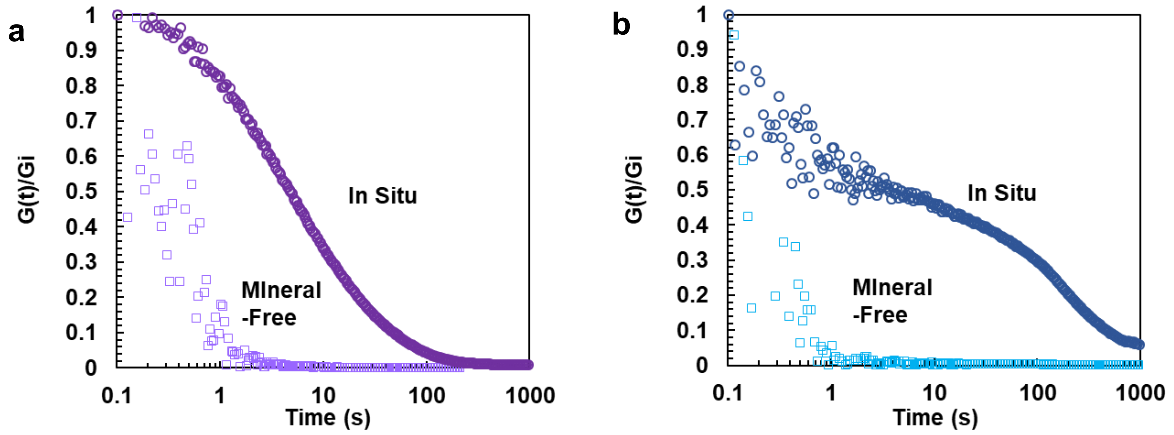

Supplementary Figure 19. Step-strain tests on Mineral-Free and In Situ samples of (a) Ni- and (b) Cu-histidine systems. The In Situ samples show slower relaxation compared to the Mineral-Free samples.

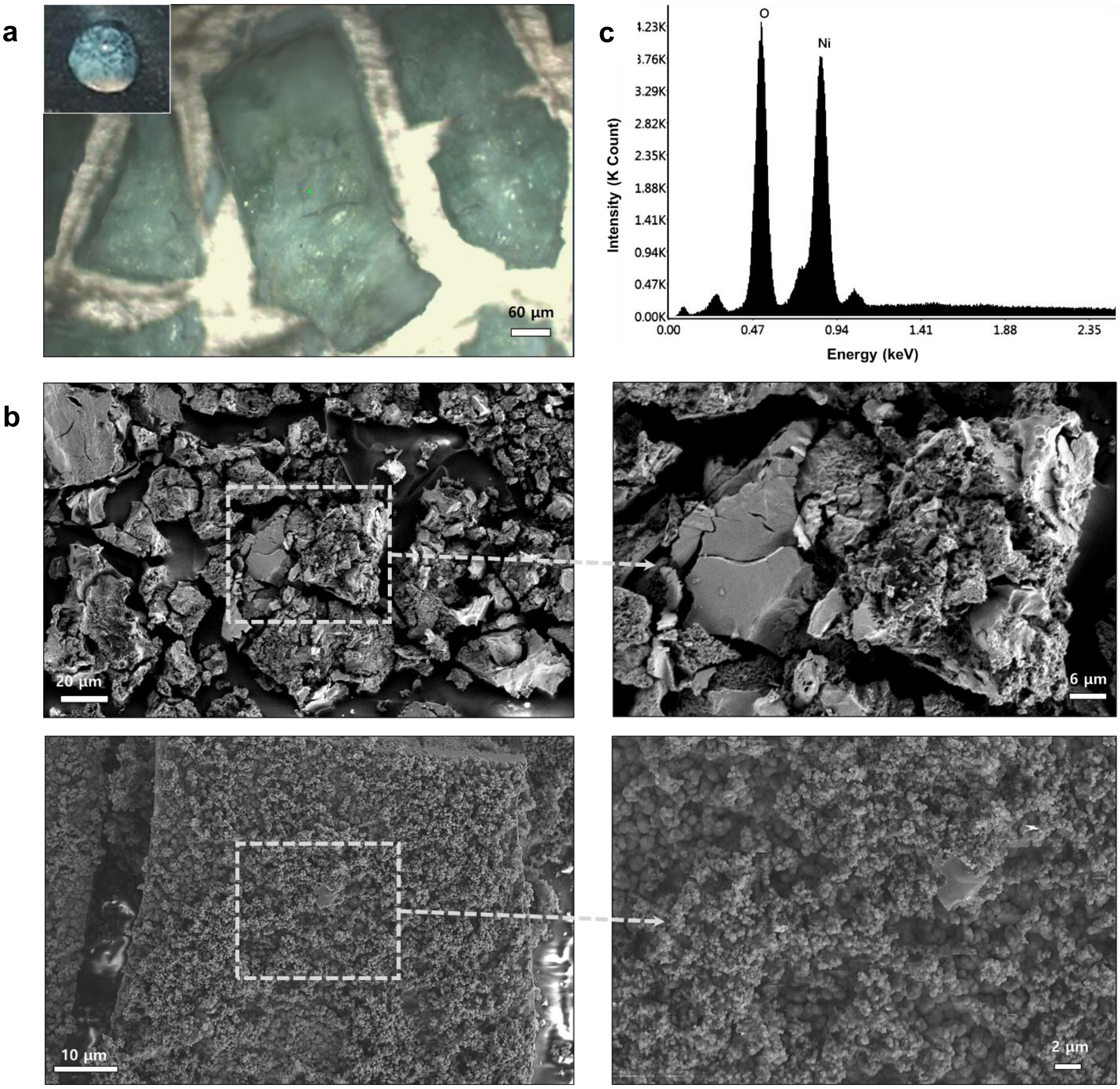

Supplementary Figure 20. (a) Confocal, (b) SEM images and (c) EDS of $Ni(OH)_2$ minerals grown without polymer (Mineral-Only). The precipitates were washed with water, then dried in ambient condition for 24 hours. (a) The precipitates showed the typical light-green color of theophrastite. Inset is the digital photograph of a Mineral-Only sample. (b) Ni minerals freely grow up to a ~ 100 µm size (upper row). Nanoparticles (lower row) are also observed as in Mineral-Only Fe minerals, which similarly attach together and grow into larger particles. (c) EDS also shows the essential element of O and Ni. We note that the Ex Situ samples showed visual mineral precipitation as in Mineral-Only.

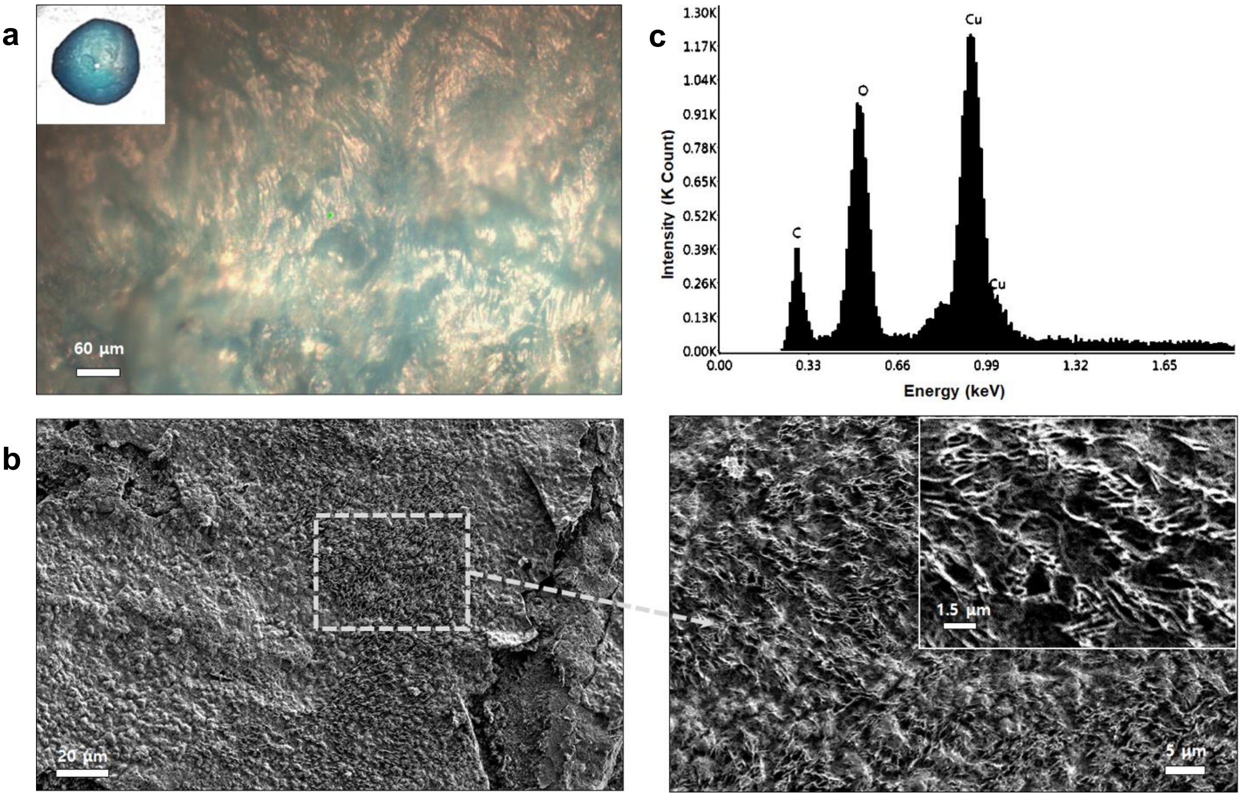

Supplementary Figure 21. (a) Confocal, (b) SEM images and (c) EDS of $Cu(OH)_2$ minerals grown without polymer (Mineral-Only). The precipitates were washed with water, then dried in ambient condition for 24 hours. (a) The precipitates showed the typical blue-green color of spertiniite. Inset is the digital photograph of a Mineral-Only sample. (b) Cu particles show platelet-like morphology composed of the wire-like surface nanostructure which resembles $Cu(OH)_2$ nanowires formed by anodization reported previously.[22] (c) EDS also shows the essential element of O and Cu. We note that the Ex Situ samples showed visual mineral precipitation as in Mineral-Only.

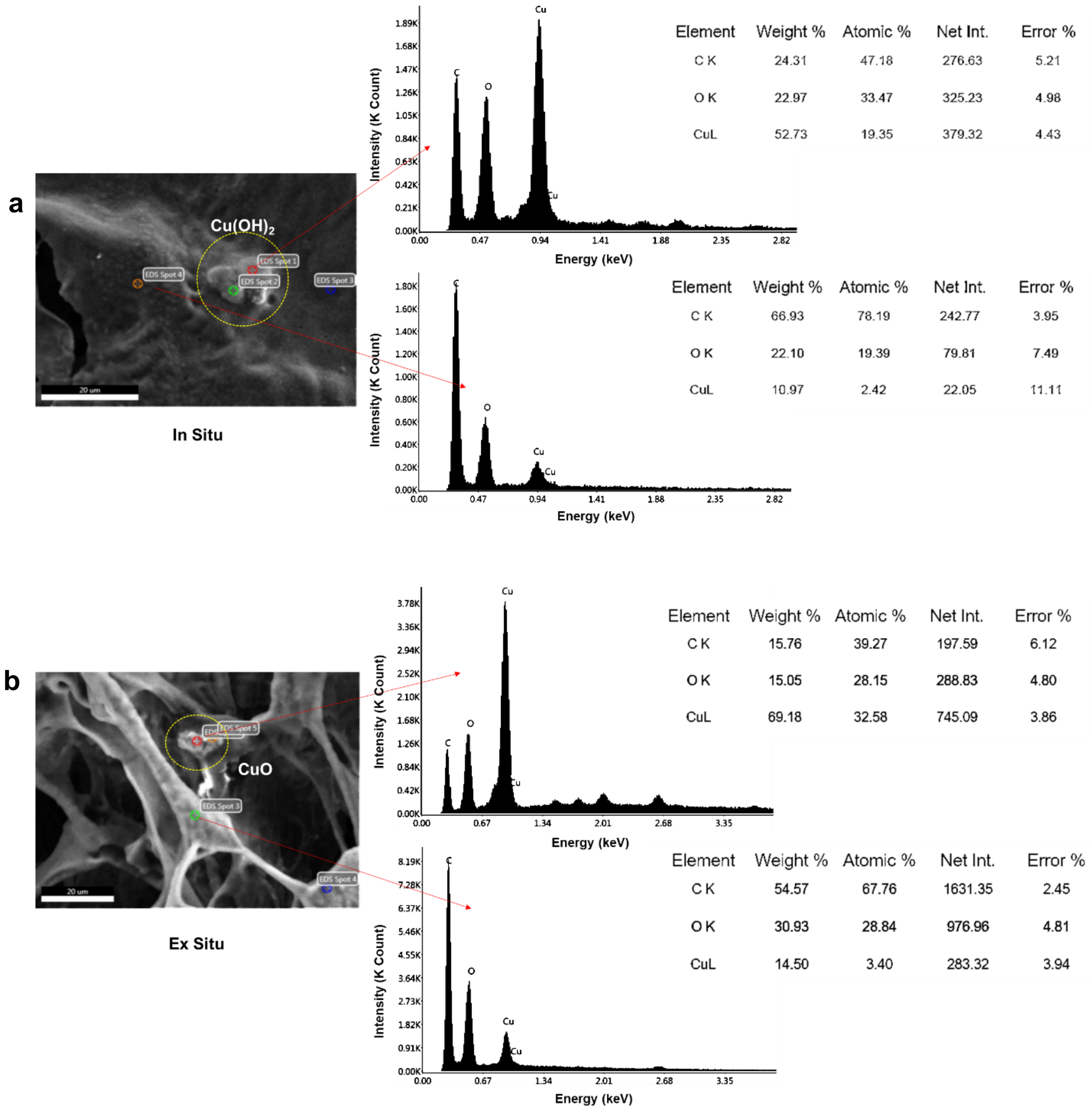

Supplementary Figure 22. EDS analysis of minerals in freeze-dried (a) In Situ and (b) Ex Situ Cu-mineral gels. Mineral particles (one representative particle circled in yellow) were detected in both gels freeze-dried for 24 hours. The exhaustive dehydration by freeze-drying allowed meaningful atomic fraction analysis, which matches the 1:2 atomic ratio between Cu and O for $Cu(OH)_2$ mineral particles, whereas the surrounding polymer matrix showed a much lower atomic fraction of Cu as expected. We note the possible inclusion of impurities such as CuO as seen in (b) likely formed from $Cu(OH)_2$ heated by laser.

Supplementary References

1. Sun, S., Gebauer, D. & Cölfen, H. Alignment of Amorphous Iron Oxide Clusters: A Non-Classical Mechanism for Magnetite Formation. *Angew. Chemie Int. Ed.* **56**, 4042–4046 (2017).

2. Altan, C. L. *et al.* Partial oxidation as a rational approach to kinetic control in bioinspired magnetite synthesis. *Chem. - A Eur. J.* **21**, 6150–6156 (2015).

3. Cornell, R. & Schwertmann, U. *The Iron Oxides: Structure, Properties, Reactions, Occurences and Uses*. (2003). doi:10.1002/3527602097.ins

4. Singh, M. *et al.* Vapour phase approach for iron oxide nanoparticle synthesis from solid precursors. *J. Solid State Chem.* **200**, 150–156 (2013).

5. Pardoe, H., Chua-anusorn, W., St. Pierre, T. G. & Dobson, J. Structural and magnetic properties of nanoscale iron oxide particles synthesized in the presence of dextran or polyvinyl alcohol. *J. Magn. Magn. Mater.* **225**, 41–46 (2001).

6. Magnetite: Mineral information, data and localities. Available at: https://www.mindat.org/min-2538.html. (Accessed: 8th April 2020)

7. Maghemite: Mineral information, data and localities. Available at: https://www.mindat.org/min-2533.html. (Accessed: 8th April 2020)

8. Fu, X. *et al.* Thermosetting solid--solid phase change materials composed of poly (ethylene glycol)-based two components: flexible application for thermal energy storage. *Chem. Eng. J.* **291**, 138–148 (2016).

9. Ilavsky, J. *et al.* Ultra-small-angle X-ray scattering at the Advanced Photon Source. *J. Appl. Crystallogr.* **42**, 469–479 (2009).

10. Fournet, G. & Guinier, A. Small angle scattering of X-rays. *Transl. by Walker, CB Yudowitch, KL New York John Wiley Sons* 7–78 (1955).

11. Beaucage, G. Approximations Leading to a Unified Exponential/Power-Law Approach to Small-Angle Scattering. *J. Appl. Crystallogr.* **28**, 717–728 (1995).

12. Payne, A. R. The dynamic properties of carbon black loaded natural rubber vulcanizates. Part II. *J. Appl. Polym. Sci.* **6**, 368–372 (1962).

13. Payne, A. R., Whittaker, R. E. & Smith, J. F. Effect of vulcanization on the low-strain dynamic properties of filled rubbers. *J. Appl. Polym. Sci.* **16**, 1191–1212 (1972).

14. Li, Q., Barrett, D. G., Messersmith, P. B. & Holten-Andersen, N. Controlling hydrogel mechanics via bio-inspired polymer-nanoparticle bond dynamics. *ACS Nano* **10**, 1317–1324 (2016).

15. Zhukhovitskiy, A. V. *et al.* Highly branched and loop-rich gels via formation of metal-organic cages linked by polymers. *Nat. Chem.* **8**, 33–41 (2016).

16. Zhukhovitskiy, A. V. *et al.* Polymer Structure Dependent Hierarchy in PolyMOC Gels. *Macromolecules* **49**, 6896–6902 (2016).

17. Holten-Andersen, N. *et al.* pH-induced metal-ligand cross-links inspired by mussel yield self-healing polymer networks with near-covalent elastic moduli. *Proc. Natl. Acad. Sci. U. S. A.* **108**, 2651–5 (2011).

18. Barrett, D. G. *et al.* PH-based regulation of hydrogel mechanical properties through mussel-inspired chemistry and processing. *Adv. Funct. Mater.* **23**, 1111–1119 (2013).

19. de Faria, D. L. A., Venâncio Silva, S. & de Oliveira, M. T. Raman microspectroscopy of some iron oxides and oxyhydroxides. *J. Raman Spectrosc.* **28**, 873–878 (1997).

20. Hanesch, M. Raman spectroscopy of iron oxides and (oxy)hydroxides at low laser power and possible applications in environmental magnetic studies. *Geophys. J. Int.* **177**, 941–948 (2009).

21. Kim, S., Peterson, A. M. A. M. & Holten-Andersen, N. Enhanced Water Retention Maintains Energy Dissipation in Dehydrated Metal-Coordinate Polymer Networks: Another Role for Fe-Catechol Cross-

Links? *Chem. Mater.* **30**, 3648–3655 (2018).

22. Ming, H. *et al.* Electrochemical fabrication of Cu(OH)2 and CuO nanostructures and their catalytic property. *J. Cryst. Growth* **327**, 251–257 (2011).
